# Enhancing the yield, quality, and potassium use efficiency of direct-seeded hybrid *indica* rice through wheat-straw returning combined with potassium fertilizer application

**DOI:** 10.3389/fpls.2026.1839324

**Published:** 2026-05-13

**Authors:** Yuanyuan Sun, Xinghai Huang, Ruijie Li, Tao Lu, Ailing Wang, Mingming Hu, Jiarui Ni, Yong Zhao, Lin Feng, Qin Liao, Zhonglin Wang, Zhiyuan Yang, Jun Ma, Yongjian Sun

**Affiliations:** 1 Crop Ecophysiology and Cultivation Key Laboratory of Sichuan Province, Sichuan Agricultural University, Chengdu, China; 2 Sichuan Climate Center, Sichuan Meteorological Bureau, Chengdu, China; 3 Yibin Nanxi Agriculture, Animal Husbandry and Fishery Science and Technology Extension Station, Yibin Nanxi Agricultural and Rural Bureau, Yibin, China; 4 Hejiang Modern Agricultural Development Promotion Center, Hejiang Agricultural and Rural Bureau, Hejiang, China

**Keywords:** direct-seeded rice, K application, K use efficiency, rice quality, wheat-straw returning

## Abstract

**Introduction:**

Previous studies have shown that straw returning combined with potassium (K) fertilization improves grain yield and K use efficiency (KUE) in transplanted rice. However, in rice–wheat rotation systems, the synergistic effects of wheat-straw returning and optimized K fertilization on yield, rice quality, and KUE of direct-seeded rice remain poorly understood. In particular, the relationships among photosynthetic material production, source–sink coordination, and K balance, in relation to grain yield and rice quality, have not been clearly elucidated.

**Methods:**

A two-factor field experiment was conducted using super hybrid *indica* rice Fyou 498 to investigate the combined effects of wheat-straw management and K fertilization on yield, rice quality, and KUE in mechanically direct-seeded rice. Two straw treatments were set: no wheat-straw returning (M_0_) and wheat-straw returning (M_1_), with each straw treatment crossed with five elemental K application rates: 0, 62.5, 125, 187.5, and 250 kg K ha^-1^.

**Results:**

Wheat-straw returning showed a statistically significant effect. Compared with M_0_, M_1_ significantly increased the photosynthetic potential from jointing to full heading by 1.83%–22.34%, improved postheading dry matter accumulation by 2.06%–36.56%, and increased yield by 1.06%–14.36%. Under M_1_ combined with 125 kg K ha^-1^, dry matter accumulation increased by 9.76%–86.53%, K translocation amount and contribution rate stem sheath (leaf) increased by 0.78%–38.34%, yield improved by 4.79%–19.95%, KUE increased by 3.61–15.15 kg kg^-1^, grain chalkiness reduced by 0.03%–2.97%, taste value increased by 0.64–5.14, and soil K depletion was reduced by 39.25–52.13 kg ha^-1^. Notably, correlation analysis showed that an increase in the contribution rate of K translocation from the stem sheath from full heading to maturity (*r* = 0.50^*^–0.96^**^) is a key pathway for achieving synergistic high yield, high quality, and high KUE in direct-seeded rice under combined wheat-straw returning and K fertilizer application.

**Discussion:**

This optimized management strategy of wheat-straw incorporation (fresh straw incorporation rate: 5,050–5,440 kg ha^-1^, straw moisture content: 28.9%–30.1%) combined with 125 kg K ha^-1^ represents a promising approach for sustaining rice production in rice–wheat rotation systems under humid subtropical monsoon pedoclimatic conditions, delivering synergistic improvements in grain yield, rice quality, resource-use efficiency, and economic benefits for mechanically direct-seeded rice.

## 1 Introduction

Meeting the food demand of the rapidly growing global population requires an increase in grain yield per unit area of cultivated land (Fu et al., 2024). Rice–wheat rotation is an effective approach to increasing the annual multiple cropping index and maximizing cultivated land utilization for higher grain production (Wen et al., 2024). China’s rice–wheat rotation systems produce 8.78 × 10^8^ tonnes of straw annually as a major agricultural by-product (Shao et al., 2023). Moderate straw return can improve soil properties by optimizing soil structure and enhancing soil water retention capacity. Straw contains abundant nitrogen (N), phosphorus (P), and potassium (K), and its return to the field helps maintain soil fertility, reduce synthetic fertilizer input, and support long-term agricultural sustainability (King and Blesh, 2018; Zhao et al., 2020; Li et al., 2023). Consistent with these benefits, existing studies have confirmed that annual straw incorporation at varying rates significantly improves soil microbial activity, crop yield, and quality in rice–wheat rotation systems. Furthermore, long-term combined incorporation of straw and organic fertilizer has been shown to advance agricultural sustainability by enhancing crop productivity and reducing environmental pollution (Xu et al., 2016; Li et al., 2025; Wu et al., 2026). However, with increasing crop yield, the residual amount of straw in fields has increased significantly, and excessive wheat-straw return poses a series of nonnegligible challenges (Beant Singh et al., 2014; Ma et al., 2025). Straw decomposition not only drives the leaching of soil base cations and ultimately decreases soil pH via the organic acids it releases (Liang et al., 2023) but also causes soil microorganisms to preferentially utilize soil inorganic N, creating competition with crops for N uptake, poisoning roots, forms a favorable microenvironment in topsoil for the survival of pathogens and pests, and ultimately reducing crop yield (Shan et al., 2021; Pingthaisong et al., 2024; Wen et al., 2024).

As a core strategic resource for national food security, China’s potash supply has long been highly import-dependent, with annual imports stabilizing at above nine million tonnes (Matheson et al., 2025). However, existing studies investigating the effects of nutrients on grain yield, rice quality and nutrient utilization characteristics have predominantly focused on N regulation under single straw return (Li et al., 2021; Lyu et al., 2021; Liu et al., 2024), and insufficient attention has been paid to the efficient utilization of K. K released from straw incorporation and applied available K fertilizer are two core sources governing K uptake and utilization in rice (Ye et al., 2020; Huo et al., 2024). Numerous studies have demonstrated that K fertilizer application improves the grain yield of transplanted rice by increasing effective panicle number, grains per panicle, grain-filling rate, and 1,000-grain weight (Bassuony, 2019; Zhang et al., 2021a; Fan et al., 2023). Existing studies have shown that the effect of K fertilization on rice quality follows a single-peak curve: an appropriate K application rate effectively improves overall rice quality, whereas insufficient K supply constrains the realization of rice’s inherent genetic quality potential, and excessive K application reduces brown rice, milled rice, and head rice rates while increasing the proportion of chalky grains. Excessive K fertilization also promotes protein accumulation in rice and decreases taste value (Chen et al., 2023). Furthermore, overapplication of K induces excessive K uptake by rice plants, which reduces K translocation efficiency, inhibits the synergistic improvement of grain yield, and ultimately leads to a low yield–cost ratio (Ye et al., 2020). However, no consensus has been reached regarding the effects of supplementary K application on yield formation, rice quality, and KUE pathways in direct-seeded rice.

As the dominant planting systems for rice (*Oryza sativa* L.) in China, manual transplanting and mechanical transplanting have long been the core focus of research and development on high-yield and high-efficiency cultivation techniques (Lyu et al., 2021; Guo et al., 2023b; Sun et al., 2023). Direct-seeded rice is a simple planting method that eliminates various production steps (such as seedling nursery, seedling lifting, transportation, and transplanting), greatly improving production efficiency and fitting well with the planting schedule of the rice–wheat rotation system (Sun et al., 2024). However, the negative effects of straw return combined with inappropriate K fertilizer management constrain the widespread adoption of direct-seeded rice (Tian et al., 2022), and current research on key cultivation techniques for achieving high yield and high efficiency in direct-seeded rice remains largely insufficient. In particular, one critical question remains unanswered: Can the interaction between straw return and K fertilizer application synergistically improve grain yield, rice quality, and KUE in direct-seeded hybrid *indica* rice while effectively alleviating soil K deficit? Further investigation is therefore required to validate the efficacy of this interaction and clarify its practical implications for rice production.

Therefore, this study aimed to investigate the interactive effects of wheat straw returning and K application on grain yield, rice quality, and KUE of direct-seeded *indica* rice in the rice–wheat rotation system. The specific objectives of this study were to: (i) examine how different combinations of wheat-straw returning and K fertilization affect grain yield, yield components, and key quality parameters of direct-seeded *indica* rice; (ii) evaluate the combined effect of the two practices on K accumulation, translocation efficiency, and KUE, and determine whether this interaction can offset the low K efficiency caused by excessive K application and alleviate soil K deficit in paddy fields; (iii) identify the optimal combination of wheat-straw returning and K fertilization based on the relationship to yield, quality, and KUE, thereby providing technical support for synergistic yield increase, quality improvement, and efficient K utilization in direct-seeded rice production.

## 2 Materials and methods

### 2.1 Test site and materials

The 2-year field experiments were conducted during 2021–2022 on rice–wheat rotation plots at the Modern Agricultural Base of Sichuan Agricultural University in Chongzhou, Sichuan (30°33′N, 103°38′E; 520.6 m altitude). Different plots were used in each year of the 2-year experiment, with an approximate distance of 50 m between plots. The site has a subtropical monsoon humid climate, and the average temperature, sunshine hours, and rainfall during the rice growing season (May to September) were 21.62 °C, 763.12 h, and 784.26 mm in 2021 and 22.05 °C, 721.03 h, and 803.61 mm in 2022, respectively. The test cultivar was Super Rice Fyou 498, an *indica*-type three-line hybrid with an average growth duration of 148.2 days. The plow layer soil (0–20 cm) was classified as sandy loam, with a particle composition of 47% sand, 34% clay, 19% silt, and a bulk density of 1.26 g cm^-3^; its basic physicochemical properties are listed in **Table 1**.

**Table 1:** Soil physicochemical properties and wheat-straw nutrient content.

Year	Organic matter (g kg^-1^)	Organic carbon (g kg^-1^)	Total N (g kg^-1^)	Effective soil nutrients (mg kg^-1^)			pH	Nutrient content of wheat-straw (g kg^-1^)		
				N	P	K		N	P	K
2021	14.71	8.54	1.60	86.45	22.58	100.31	6.47	9.74	0.37	11.69
2022	15.02	8.73	1.64	94.38	23.61	95.08	6.52	8.88	0.36	12.10

### 2.2 Experimental design

The experiment employed a split-plot design with two factors. The main plots consisted of no straw returning (M_0_) and wheat-straw returning (M_1_). The subplots comprised five elemental K fertilizer application rates (K chloride [KCl], 49.8% elemental K content): 0 (K_0_), 62.5 (K_62.5_), 125 (K_125_), 187.5 (K_187.5_), and 250 (K_250_) kg K ha^-1^. Each treatment received N fertilizer (urea, 46% N content) applied at 135 kg N ha^-1^, according to a base fertilizer (1 day before sowing) and tiller fertilizer (22 days after sowing) at a ratio of 7:3. P fertilizer (calcium superphosphate, 12% P_2_O_5_ content) was applied at 90 kg P_2_O_5_ ha^-1^ as a one-time basal application. K fertilizer was applied according to a base, tiller, and panicle fertilizer schedule (70 days after sowing) at a ratio of 5:2:3.

The day after the wheat harvest, all the wheat-straw was crushed to 3–5 cm and incorporated into the plow layer soil at a depth of 5–10 cm using a tiller, with 5,050 kg ha^-1^ (28.9% moisture content of straw) and 5,440 kg ha^-1^ (30.1% moisture content of straw) fresh weight of straw returned on 28 April in 2021 and 30 April in 2022, respectively. After radicle emergence in soaked rice seeds, shade drying without staining was performed. The seeds were sown on 5 May in 2021 and 7 May in 2022 using the direct-seeding machine of Shanghai Star Modern Agricultural Machinery Co. Ltd., Shanghai, China. (2BDXS-10CP), with a row spacing of 25.0 cm × 20.0 cm at three to four grains per hole, and harvesting was performed on 22 September in 2021 and 20 September in 2022. With three replicates, totaling 30 plots (2 main plots × 5 subplots × 3 replicates = 30 plots; 18.7 m^2^ per plot; dimensions: 3.4 m × 5.5 m), ridges covered with plastic film were installed between plots to prevent cross-plot water or fertilizer exchange. The high-efficiency irrigation technique described by Sun et al. (2012) was used, with a water meter ensuring the same level in each plot. Chemical pesticides were used for weeding and pest control to prevent yield loss and experimental errors.

### 2.3 Measurement items and methods

#### 2.3.1 Dry matter accumulation and translocation

Using the method of Guo et al. (2023a), five representative plants were sampled from holes in each plot based on the average number of tillers at the tillering (45 days after sowing), jointing, full-heading, and maturity stages. Each plant was divided into stem sheaths, leaves, panicles, and roots, and then placed in a constant-temperature drying oven at 105°C for 45 min and subsequently at 80°C to a constant weight.

#### 2.3.2 Leaf area index

Five representative plants were sampled in each plot based on the average number of tillers at the jointing and full-heading stages. The green leaf area was measured using the CID-203 leaf area meter (CID Company, Delaware, USA), and the leaf area index (LAI) was calculated using the method reported by Ran et al. (2023).

#### 2.3.3 Net photosynthetic rate

Using the method of Sun et al. (2012), five holes representing plants with average tillers were sampled from each plot, and the net photosynthetic rate (*P*_n_) of five flag leaves of the main-stem were measured using the Li-6400 photosynthesis system (Li-COR Inc., Nebraska, USA) between 10:00 and 11:30 on sunny days (0, 15, and 30 days after the full-heading stage). The leaf chamber conditions were as follows: CO_2_ concentration of 400 μmol mol^-1^, temperature of 30 °C, and light intensity of 1200 μmol m^-2^ s^-1^.

#### 2.3.4 K utilization

As mentioned in **Section 2.3.1**, the dried stem sheaths, leaves, panicles, and roots were crushed through an 80-mesh sieve and decocted with H_2_SO_4_–H_2_O_2_. Using the method of Zhang et al. (2021b), K content in stem sheaths, leaves, panicles, and roots was quantified by flame photometry (FP640, Shanghai Yidian).

#### 2.3.5 Grain yield and its components

At maturity, 50 holes representative plants from each plot were surveyed. The number of effective panicles was counted for each plant, and the mean was calculated. Ten plants with the average number of effective panicles were then selected from each plot, and spikelet number per panicle, filling rate, and 1,000-grain weight were calculated. Each plot was harvested over a 12.0-m^2^ area to calculate the yield (calculated at 13.5% standard moisture content).

#### 2.3.6 Straw decay rate

Following the nylon mesh bag method of Ocio et al. (1991), intact crop straw samples of uniform thickness and length were selected and cut into 3–5-cm segments. Exactly 30.0 g of cut straw was weighed into each 40-mesh nylon mesh bag (30 cm × 35 cm), and the bags were buried at a depth of 5–10 cm in the plow layer. Five random bags were retrieved from each plot at the tillering, jointing, full-heading, and maturity stages. After retrieval, the straw was rinsed thoroughly with tap water until the water turned colorless and then oven-dried at 80 °C to constant weight.

#### 2.3.7 Soil available K content

Soil samples (0–20 cm depth) were collected with a soil auger at the tillering, jointing, full-heading, and maturity stages. After air-drying, thorough grinding, and sieving through an 80-mesh sieve, 5.0 g of processed soil was extracted with 1.0 mol L^-1^ ammonium acetate. Available K content was determined by flame photometry following the method of Zhang et al. (2021).

#### 2.3.8 Rice quality

Following the method of Sun et al. (2025), 1,000 g of seeds were collected from each plot after harvest, naturally air-dried for 3 months, and air-screened under constant airflow to remove impurities for subsequent quality analysis. For brown rice rate and head milled rice rate determination, 250.0 g of cleaned grains were milled twice with a huller and rice mill, then separated into broken and intact grains. Both traits were calculated as the percentage of the initial sample weight. A total of 1,000 intact head rice grains were randomly selected for digital imaging, and this procedure was repeated three times. The JMWT-12 image analysis software (Dongfujiuheng Instrument Technology Co. Ltd., Beijing, China) was used to determine chalkiness rate and chalkiness degree according to the method of Guo et al. (2023b). Following the method of Shi et al. (2022) with improvements, rice taste value was determined using a Satake STA1A Rice Taste Analyzer (Satake, Hiroshima, Japan); 30.0 g of milled rice was placed in a stainless-steel tank and soaked for 30.0 min at a rice-to-water ratio of 1:1.3. After wrapping the tank mouth with filter paper, the sample was steamed for 30.0 min in an electric steam cooker and cooled for 2.0 h. Finally, 7.0 g of steamed rice was pressed into a rice cake at 25°C using a dedicated pressing instrument for taste assessment. The taste value of each sample was scored on a scale with a minimum of 100. The higher the taste value, the better the rice eating quality.

### 2.4 Indicator calculation

The following indicators were calculated according to the methods of Ocio et al. (1991); Singh et al. (2018); Han et al. (2021), Mohapatra et al. (2023), and Sun et al. (2024).

1. Stem-sheath matter translocation rate (%) = ([Dry weight of stem sheath at full-heading stage] – [Dry weight of stem sheath at maturity stage])/Dry weight of stem sheath at full-heading stage × 100.
2. Stem-sheath matter translocation contribution rate (%) = ([Dry weight of stem sheath at full-heading stage] – [Dry weight of stem sheath at maturity stage])/Dry grains weight × 100.
3. Population growth rate (g m^-2^ day^-1^) = (W_2_–W_1_)/(t_2_–t_1_), where W_1_ and W_2_ represent the dry matter weights in the early and late growth stages, respectively; *t*_1_ and *t*_2_ represent the times of the early and late growth stages, respectively.
4. Population photosynthetic potential (× 10^4^ m^2^ day ha^-1^) = 1/2 (L_1_ + L_2_) × (t_2_–t_1_), L_1_ and L_2_ represent the green leaf areas in the early and late growth stages, respectively; *t*_1_ and *t*_2_ represent the times of the early and late growth stages, respectively.
5. K translocation amount (kg ha^-1^) = (K uptake in stem sheaths or leaves at full-heading stage) − (K uptake in stem sheaths or leaves at maturity stage).
6. K translocation contribution rate (%) = (K translocation amount/K accumulation in grains at maturity) × 100.
7. K agronomic efficiency (kg kg^-1^) = ([Yield of K-fertilized plot] – [Yield of K-free plot])/K application rate.
8. K physiological efficiency (kg kg^-1^) = ([Yield of K-fertilized plot] – [Yield of K-free plot])/([K uptake in K-fertilized plot] – [K uptake in K-free plot]).
9. Straw decay rate (%) = (W_0_ − W_i_)/W_0_ × 100%, where *W*_0_ and *W_i_* represent the dry matter weights before straw returning and at the *i*th sampling after straw returning, respectively.
10. K surplus or deficit amount (kg ha^-1^) = Total K input from K fertilizer + K input from straw returning − Total K uptake by rice plant.
11. K surplus or deficit ratio (%) = K surplus or deficit amount/([K fertilizer rate + straw K input]) × 100.

### 2.5 Determine the optimal treatment

Using principal component analysis and membership function to comprehensively evaluate the effects of different treatments on grain yield, KUE, and rice quality to determine the optimal treatment (Song et al., 2024).

1. Principal component analysis: Extract principal components based on the condition that the eigenvalues are greater than 1 and the cumulative contribution rate of the principal components is greater than 85%.
2. Membership function value: μ(X_i_) = (X_i_ − X_min_)/(X_max_ − X_min_); i = 1, 2, 3, …, n. Among them, *X_i_* represents the *i*th comprehensive indicator, *X*_min_ represents the minimum value of the *i*th comprehensive indicator, and *X*_max_ represents the maximum value of the *i*th comprehensive indicator.
3. Weight: ${\text{W}}_{i}={\text{P}}_{\text{i}}/\sum_{\text{i}=1}^{\text{n}}{\text{P}}_{\text{i}}$, W_i_ represents the weight of the *i*th principal component, and *P_i_* is the contribution rate of the *i*th comprehensive indicator.
4. Comprehensive scores of each treatment: $\text{D}=\sum_{\text{i}}^{n}\left(\mu \left[{\text{X}}_{\text{i}}\right]\times {\text{W}}_{\text{i}}\right)$

### 2.6 Data analysis

Data were analyzed using Microsoft Excel 2024 for data management and preliminary calculations; SPSS 24.0 (IBM Corp., Armonk, NY, USA) for analysis of variance (ANOVA), testing of interaction effects between year and treatments, and multiple comparisons via the least significant difference (LSD) method; and Origin 2021 (OriginLab Corp., Northampton, MA, USA) for graphing, correlation analysis, and principal component analysis (PCA). ANOVA results showed that the interaction effects between years and straw treatments, as well as between years and K treatments, on grain yield, yield components, and KUE of rice were all nonsignificant (*p* > 0.05) across the 2 years (**Supplementary Table 1**). Similar results were obtained for the same wheat straw returning and K treatments across different years. Unless otherwise specified, this paper focuses on analyzing the experimental results of 2022.

## 3 Results

### 3.1 Yield and its components

Wheat-straw returning, K application, and their interactive effects significantly affected grain yield (*p* < 0.05; **Table 2**). Across all experimental years, grain yield in the M_1_ treatment increased by 1.06%–14.36% to varying degrees compared with M_0_. The optimal K application rate differed across the wheat-straw returning treatments: under M_0_, the highest yield was achieved at 187.5 kg K ha^-1^. By contrast, under M_1_, the maximum yield was obtained at 125 kg K ha^-1^. The yield of the optimal combination of M_1_ and 125 kg K ha^-1^ (M_1_K_125_) was 3.56%–28.19% higher than that of all other treatments, confirming this approach as an effective strategy for achieving high yield while reducing K input. Accordingly, this combination was identified as the optimal K treatment in this experiment. Further increases in K input beyond this optimal rate caused a significant yield reduction of 3.44%–9.85%.

**Table 2:** Effects of straw returning and K application rates on grain yield and its components of direct-seeded rice.

Treatments	Effective panicles (× 10^4^ ha^-1^)	Spikelets (No. panicle)	Total spikelets (× 10^6^ ha^-1^)	Filled grains (%)	1,000-grain weight (g)	Grain yield (kg ha^-1^)	
						2021	2022
M_0_							
K_0_	189.73 d	154.18 c	292.53 d	82.45 ab	31.02 b	7,027.3 d	7,427.5 d
K_62.5_	193.93 c	159.44 b	309.20 c	81.35 c	31.56 ab	7,700.1 c	7,927.6 c
K_125_	199.90 b	162.42 ab	324.68 b	82.06 bc	31.79 a	8,484.9 b	8,378.6 bc
K_187.5_	208.63 a	166.04 a	346.40 a	83.19 a	31.81 a	8,901.2 a	8,933.8 a
K_250_	205.90 a	166.58 a	342.98 a	78.23 d	31.90 a	8,599.7 ab	8,524.7 ab
Average	199.62	161.73	323.16	81.46	31.62	8,142.6	8,238.4
M_1_							
K_0_	192.01 d	155.54 b	298.65 d	82.55 b	31.21 b	7,569.2 d	7,529.4 c
K_62.5_	198.77 c	159.62 b	317.28 c	82.67 b	31.71 ab	8,457.4 c	8,248.3 b
K_125_	211.30 a	171.46 a	362.28 a	84.43 a	31.84 ab	9,703.3 a	9,509.2 a
K_187.5_	210.73 ab	168.38 a	354.84 ab	83.25 b	31.91 a	9,369.9 ab	9,074.2 a
K_250_	206.13 b	166.78 a	343.79 b	78.38 c	31.69 ab	9,257.3 b	8,615.2 b
Average	203.79	164.36	335.37	82.25	31.67	8,871.4	8,595.3
*F-*value							
M	9.20^*^	8.66^*^	14.09^**^	5.71^*^	0.12	59.94^**^	14.47^*^
K	11.97^**^	7.42^*^	24.53^**^	6.76^*^	1.82	111.12^**^	29.65^**^
M × K	5.12^*^	2.59	14.53^**^	2.01	1.74	3.02^*^	7.04^**^

Different lowercase letters under the same straw returning method in the same column indicate significant differences at the 5% level among different K rates. K_0_, K_62.5_, K_125_, K_187.5_, and K_250_ represent the elemental K fertilizer rates: 0, 62.5, 125, 187.5, and 250 kg ha^-1^, respectively.

*M_0_*, no straw returning; *M_1_*, straw returning.

^*^ *p* < 0.05 and ^**^p < 0.01 indicate significant effects.

*F*-value, F-statistic in analysis of variance; M, wheat-straw returning treatment; K, K application; M×K, wheat-straw returning treatment and K application interaction.

Effective panicles, spikelets per panicle, filled grains, and grain weight are the main yield component factors. Wheat-straw returning and K application had significant (*p* < 0.05) or extremely significant (*p* < 0.01) effects on effective panicles, spikelets per panicle, total spikelets, and filled grains. Additionally, the two factors exhibited significant interactive effects on effective panicles (*p* < 0.05) and total spikelets (*p* < 0.01) (**Table 2**). Relative to M_0_, M_1_ increased effective panicles, spikelets per panicle, and total spikelets by 1.62%–3.87% and increased filled grains by 0.06%–2.36%. Across all combinations of wheat-straw returning and K fertilizer application, the M_1_K_125_ treatment recorded the highest values of effective panicles, total spikelets, and filled grains, enabling high yield to be maintained with reduced K fertilizer input. The 2 years of experimental results showed a consistent trend.

### 3.2 Photosynthetic properties

Photosynthetic characteristics, including photosynthetic area, net photosynthetic rate, and dynamic photosynthetic performance across different growth stages, are the core determinants of crop yield. As shown in **Table 3**, except for LAI at the jointing stage (J-LAI), wheat-straw returning, K application, and their interaction effects significantly (*p* < 0.05) or extremely significantly (*p* < 0.01) influenced LAI at full-heading stage (F-LAI), photosynthetic potential from jointing to full-heading stage (JFPP), population growth rate from jointing to full-heading stage (JFGT), and population growth rate from full-heading to maturity stages (FMGT). Relative to the M_0_ treatment, M_1_ significantly (*p* < 0.05) increased F-LAI, JFPP, JFGT, and FMGT by 2.34%–25.60%. Across all straw returning treatments, J-LAI, F-LAI, and JFPP all increased to varying degrees as the K application rate rose, with the highest values recorded at 250 kg K ha^-1^. However, the response of the population growth rate in each growth period to K application differed between straw returning treatments. Under M_0_, both JFGT and FMGT peaked at 187.5 kg K ha^-1^. Under M_1_, both traits peaked at 125 kg K ha^-1^, representing increases of 7.97%–86.53% relative to other K application rates under the same straw returning treatment.

**Table 3:** Effects of straw returning and K application rates on the photosynthetic characteristics of direct-seeded rice.

Treatments	LAI		Population photosynthetic potential from jointing to full-heading stage (×^4^ m^2^ day^-1^ ha^-1^)	Population growth rate (g m^-2^ day^-1^)	
	Jointing stage	Full-heading stage		Jointing to full-heading stage	Full-heading to maturity stage
M_0_					
K_0_	5.02 d	5.74 c	252.86 d	11.48 d	8.22 d
K_62.5_	5.62 c	6.39 b	282.24 c	13.67 c	11.17 c
K_125_	6.24 b	7.12 a	313.96 b	15.40 ab	12.46 b
K_187.5_	6.37 ab	7.32 a	321.72 ab	17.31 a	13.83 a
K_250_	6.67a	7.37 a	329.94 a	15.26 b	12.63 b
Average	5.98	6.79	300.14	14.62	11.66
M_1_					
K_0_	5.37 d	6.24 c	272.84 d	13.23 d	8.39 d
K_62.5_	6.04 c	6.95 b	305.27 c	14.46 c	11.99 c
K_125_	6.43 bc	7.35 ab	323.83 b	18.84 a	15.65 a
K_187.5_	6.59 ab	7.42 a	329.24 ab	17.45 b	14.27 b
K_250_	6.85 a	7.59 a	339.34 a	15.54 c	13.22 b
Average	6.26	7.11	314.10	15.90	12.70
*F*-value					
M	2.98	5.19^*^	19.02^**^	5.36^*^	6.90^*^
K	9.65^**^	6.87^*^	38.14^**^	11.84^**^	47.22^**^
M × K	1.49	3.87^*^	8.28^**^	4.48^*^	3.59^*^

Different lowercase letters under the same straw returning method in the same column indicate significant differences at the 5% level in different K rates. K_0_, K_62.5_, K_125_, K_187.5_, and K_250_ represent the elemental K fertilizer rates: 0, 62.5, 125, 187.5, and 250 kg ha^-1^, respectively.

*M_0_*, no straw returning; *M_1_*, straw returning.

^*^ *p* < 0.05 and ^**^P < 0.01 indicating significant effects.

*F*-value, F-statistic in analysis of variance; M, wheat-straw returning treatment; K, K application; M×K, wheat-straw returning treatment and K application interaction.

**Figure 1:**
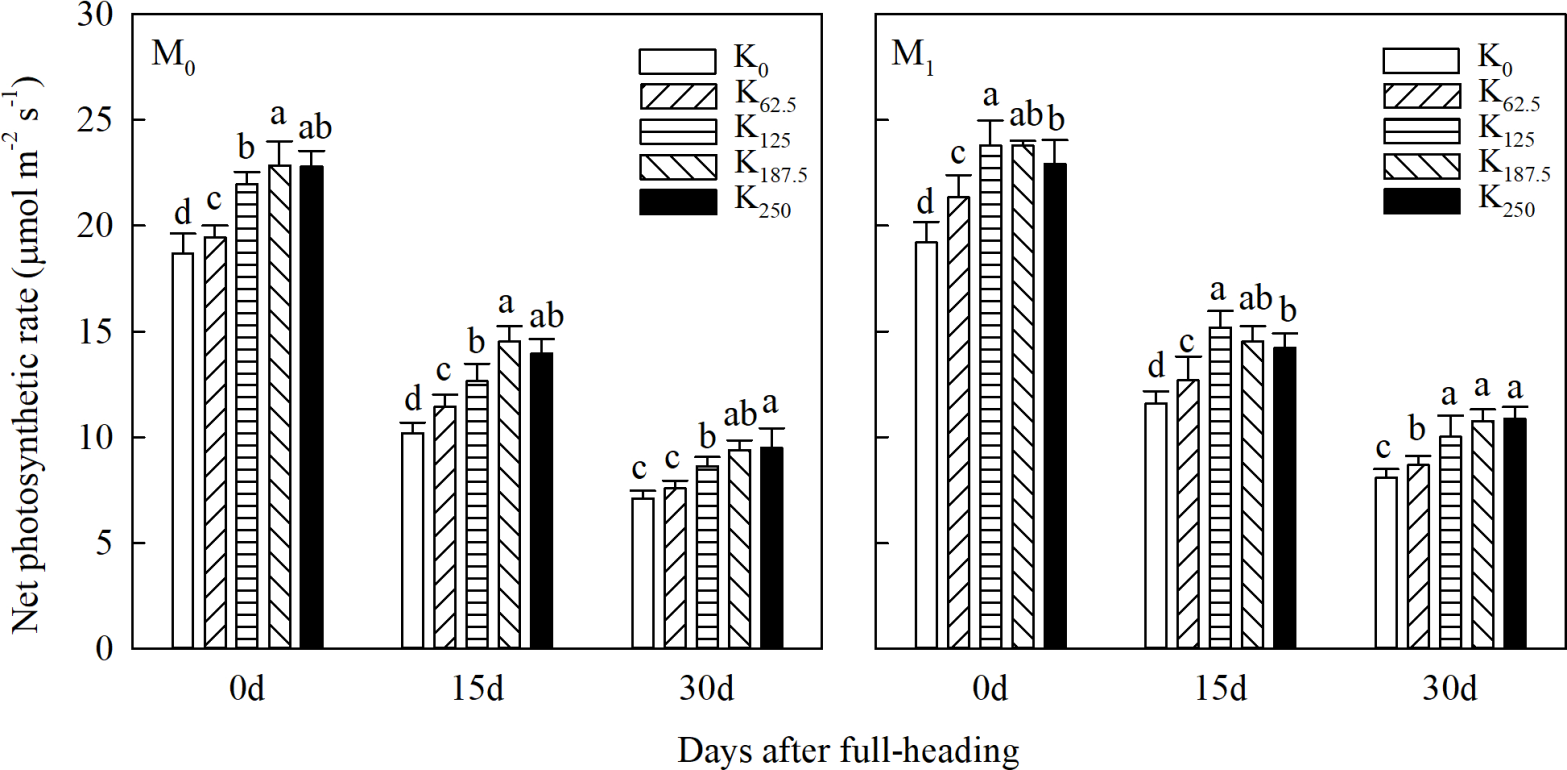
Effects of straw returning and K application rates on the net photosynthetic rate of flag leaves after full heading of direct-seeded rice. Different lowercase letters under the same straw returning method in the same column indicate significant differences at the 5% level in different K rates. M_0_, no straw returning; M_1_, straw returning. K_0_, K_62.5_, K_125_, K_187.5_, and K_250_ represent the elemental K fertilizer rates: 0, 62.5, 125, 187.5, and 250 kg ha^-1^, respectively.

Additionally, as shown in **Figure 1**, relative to the M_0_ treatment, M_1_ increased the *P*_n_ of flag leaves across all after full-heading stages by 0.43%–16.11%. Within the same straw returning treatment, *P*_n_ measured from 0 to 15 days after full heading showed a consistent trend of first increasing then decreasing as K application rates rose, indicating that excessive K input fails to further improve *P*_n_ and may even exert an inhibitory effect. Across all combinations of wheat-straw returning and K fertilizer application, the M_1_K_125_ treatment recorded the highest values, which were 0.12%–42.62% higher than those of other treatments. At 30 days after the full-heading stage, the *P*_n_ decay rate of each straw returning treatment was slower at 250 kg K ha^-1^, and it still maintained a relatively high level, leading to delayed maturity with excessive greenness.

### 3.3 Matter accumulation and translocation

The final economic yield is determined by the dry matter accumulation during the grain-filling stage of crops and the translocation efficiency of stored substances from vegetative organs to harvest organs. Wheat-straw returning, K application, and their interaction significantly (*p* < 0.05) or extremely significantly (*p* < 0.01) affected dry matter accumulation and translocation after full heading. However, the effect of wheat-straw returning on stem-sheath dry matter weight at maturity (MSW) was not significant (*p* > 0.05) (**Table 4**). Compared with the M_0_ treatment, M_1_ increased stem-sheath dry matter weight at full heading (FSW), MSW, and dry matter accumulation from full heading to maturity (FMSW) by 0.49%–36.56%. Furthermore, for the period from full heading to maturity, the stem-sheath translocation rate (EPMSS) and translocation contribution rate (CPMSS) increased by 0.82%–5.34%. Under different straw returning treatments, FSW and MSW increased gradually with increasing K application, peaking at 250 kg K ha^-1^. However, no significant differences (*p* > 0.05) were detected between the 187.5 and 250 kg K ha^-1^ treatments under M_0_, nor between the 125 and 250 kg K ha^-1^ treatments under M_1_. Furthermore, FMSW, EPMSS, and CPMSS showed a consistent trend of an initial increase followed by a decrease with increasing K application across all straw returning treatments. Across all combinations of wheat-straw returning and K application, the M_1_K_125_ treatment recorded the highest values for FMSW, EPMSS, and CPMSS, which were 0.27%–42.62% higher than those of all other treatments.

**Table 4:** Effects of straw returning and K application rates on matter accumulation and translocation in the stem sheath during the grain-filling stage of direct-seeded rice.

Treatments	FSW (kg ha^-1^)	MSW (kg ha^-1^)	FMSW (kg ha^-1^)	EPMSS (%)	CPMSS (%)
M_0_					
K_0_	5,930.6 d	4,923.4 b	2,712.6 d	16.98 d	13.56 d
K_62.5_	6,234.3 c	5,050.1 b	3,686.1 c	18.99 c	14.94 c
K_125_	6,662.7 b	5,110.9 b	3,781.8 bc	23.29 ab	18.52 b
K_187.5_	7,079.8 a	5,338.2 b	4,563.9 a	24.60 a	19.49 a
K_250_	7,182.9 a	5,617.5 a	4,167.9 b	21.79 b	18.36 b
Average	6,618.1	5,208.0	3,782.5	21.13	16.98
M_1_					
K_0_	6,060.3 c	4,947.4 d	2,768.7 d	18.36 d	14.78 c
K_62.5_	6,386.5 b	5,086.6 cd	3,956.7 c	20.35 c	15.76 c
K_125_	7,351.1 a	5,246.3 bc	5,164.5 a	28.63 a	22.13 a
K_187.5_	7,405.8 a	5,421.5 ab	4,709.1 b	26.79 a	21.87 a
K_250_	7,449.2 a	5,692.8 a	4,362.6 b	23.58 b	20.39 b
Average	6,930.6	5,278.9	4,192.3	23.54	18.99
*F*-value					
M	6.23^*^	2.34	26.30^**^	6.87^*^	6.36^*^
K	90.13^**^	28.20^**^	41.14^**^	7.94^**^	49.54^**^
M × K	14.65^**^	13.32^**^	37.37^**^	8.91^**^	5.90^*^

Different lowercase letters under the same straw returning method in the same column indicate significant differences at the 5% level in different K rates. K_0_, K_62.5_, K_125_, K_187.5_, and K_250_ represent the elemental K fertilizer rates: 0, 62.5, 125, 187.5, and 250 kg ha^-1^, respectively.

*FSW*, stem-sheath dry matter weight at the full-heading stage; *MSW*, stem-sheath dry matter weight at the maturity stage; *FMSW*, dry matter weight from the full-heading stage to maturity stage; *EPMSS*, stem-sheath matter translocation rate from the full-heading stage to maturity stage; *CPMSS*, stem-sheath matter translocation contribution rate from the full-heading stage to maturity stage; *M_0_*, no straw returning; *M_1_*, straw returning.

^*^ *p* < 0.05 and ^**^p < 0.01 indicate significant effects.

*F*-value, F-statistic in analysis of variance; M, wheat-straw returning treatment; K, K application; M×K, wheat-straw returning treatment and K application interaction.

### 3.4 K accumulation, translocation, and utilization efficiency

**Figure 2:**
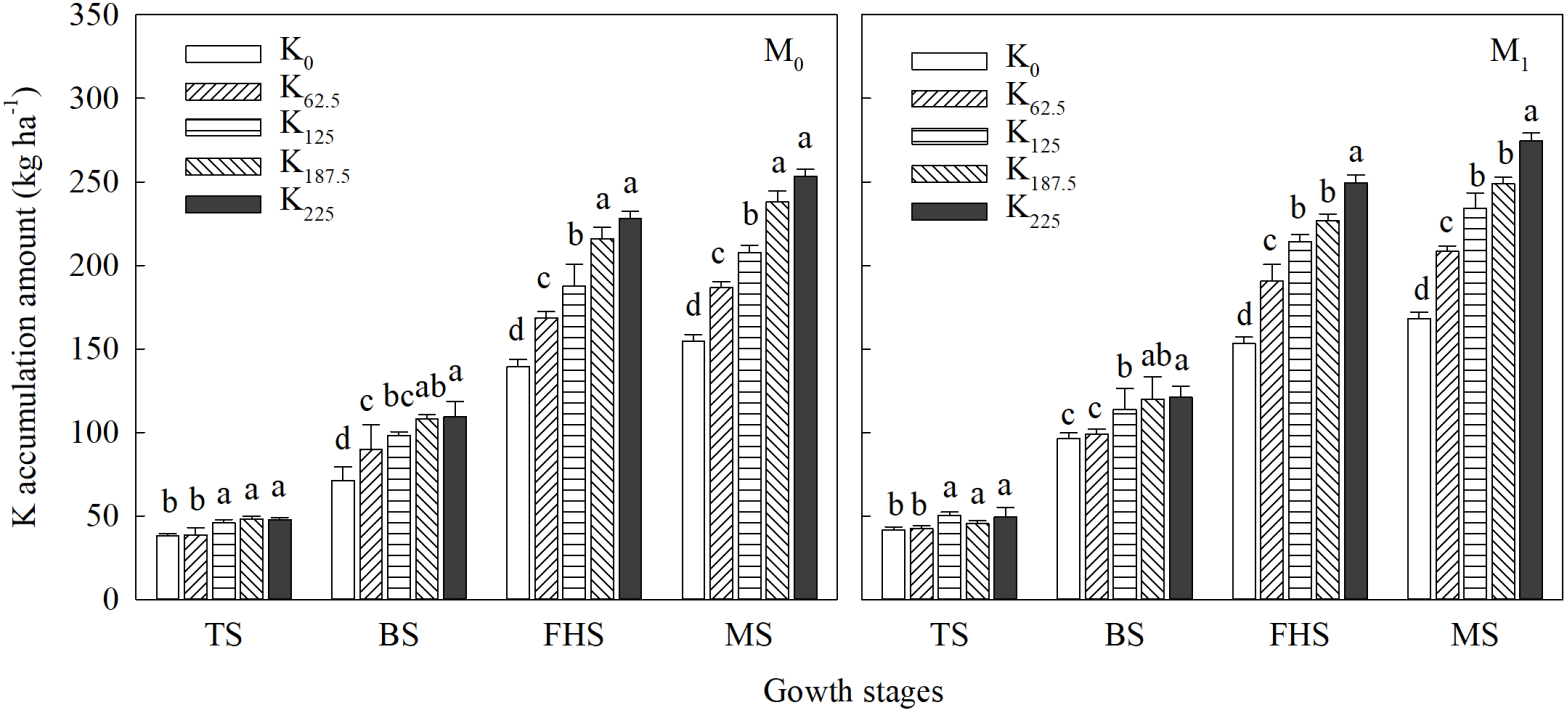
Effects of straw returning and K application rates on K accumulation at the main growth stages of direct-seeded rice. Different lowercase letters under the same straw returning method in the same column indicate significant differences at the 5% level in different K rates. M_0_, no straw returning; M_1_, straw returning; TS, tillering stage; BS, jointing stage; FHS, full-heading stage; MS, maturity stage. K_0_, K_62.5_, K_125_, K_187.5_, and K_250_ represent the elemental K fertilizer rates: 0, 62.5, 125, 187.5, and 250 kg ha^-1^, respectively.

Balanced K nutrition is the foundation for high and stable crop yields. As shown in **Figure 2**, compared with M_0_, M_1_ increased plant K accumulation by 4.62%–19.67% at different growth stages. Under different straw returning treatments, K accumulation at jointing, full heading, and maturity increased with rising K application, peaking at 250 kg K ha^-1^ (1.39%–64.00% higher than all other K treatments). The excessively high K accumulation under 250 kg K ha^-1^ at full heading and maturity indirectly suggests that this application rate impairs dry matter translocation during the grain-filling stage (**Table 4**). As shown in **Table 5**, wheat-straw returning, K application, and their interaction exerted significant (*p* < 0.05) or highly significant (*p* < 0.01) effects on K translocation amount and K translocation contribution rate in stem sheaths (leaves) during the grain-filling stage, as well as K agronomic efficiency (KAE) and K physiological efficiency (KRE). Compared with the M_0_ treatment, M_1_ increased K translocation amount and contribution rate in stem sheaths (leaves), KAE, and KRE by 0.78%–38.34%, 1.86–2.48 kg kg^-1^, and 1.93–2.21 kg kg^-1^, respectively. The results indicate that although excessive K application promotes K accumulation in plants (**Figure 2**), it impedes K translocation from vegetative organs to grains, ultimately leading to a significant decrease in both KAE and KRE. Under identical wheat-straw returning treatments, K translocation amount in stem sheaths (leaves) increased with higher K application, with no significant (*p* > 0.05) differences among 187.5–250 kg K ha^-1^. By contrast, K translocation contribution rate in stem sheaths (leaves), KAE, and KRE all first increased and then decreased with rising K application across all straw returning treatments. Across all combinations of wheat-straw returning and K application, M_1_K_125_ recorded the highest K translocation contribution rate in stem sheaths (leaves), KAE, and KRE, which were 2.07%–13.96%, 3.61–9.58 kg kg^-1^, and 7.48–15.15 kg kg^-1^ higher than all other treatments, respectively.

**Table 5:** Effects of straw returning and K application rates on K translocation during the grain-filling stage and KUE of direct-seeded rice.

Treatments	K translocation amount (kg ha^-1^)		K translocation contribution rate (%)		K agronomic efficiency (kg kg^-1^)		K physiological efficiency (kg kg^-1^)	
	Stem sheath	Leave	Stem sheath	Leave	2021	2022	2021	2022
M_0_								
K_0_	43.81 d	24.04 c	18.51 c	36.53 c	–	–	–	–
K_62.5_	56.98 c	27.21 b	21.08 b	37.84 bc	8.97 a	6.67 a	20.40 b	19.01 b
K_125_	63.86 b	29.50 b	21.53 b	40.58 ab	9.72 a	6.34 a	22.47 a	22.00 a
K_187.5_	72.29 a	33.92 a	23.84 a	42.84 a	8.33 a	6.69 a	23.19 a	22.97 a
K_250_	73.07 a	36.04 a	21.66 b	39.60 b	5.24 b	3.66 b	15.91 c	12.94 c
Average	62.00	30.14	21.50	39.48	8.06	5.84	20.49	19.23
M_1_								
K_0_	46.36 c	29.12 b	21.83 d	40.07 c	–	–	–	–
K_62.5_	60.57 b	32.26 b	23.41 d	42.08 c	11.84 b	9.59 b	21.55 b	20.31 b
K_125_	73.64 a	40.62 a	35.79 a	50.53 a	14.23 a	13.20 a	29.80 a	29.55 a
K_187.5_	75.71 a	40.69 a	33.72 b	47.52 b	8.00 c	6.87 c	22.32 b	21.50 b
K_250_	75.88 a	40.78 a	30.86 c	41.48 c	5.63 d	3.62 d	16.01 c	14.40 c
Average	66.23	36.89	29.92	44.34	9.93	8.32	22.42	21.44
*F*-value								
M	24.23^**^	6.28^*^	9.02^**^	5.91^*^	19.90^**^	8.82^*^	6.39^*^	21.52^**^
K	61.15^**^	18.20^**^	26.04^**^	17.25^**^	44.28^**^	18.31^**^	99.18^**^	76.04^**^
M × K	16.51^**^	4.93^*^	6.79^**^	5.15^*^	9.39^**^	4.87^*^	9.26^**^	18.53^**^

Different lowercase letters under the same straw returning method in the same column indicate significant differences at the 5% level in different K rates. K_0_, K_62.5_, K_125_, K_187.5_, and K_250_ represent the elemental K fertilizer rates: 0, 62.5, 125, 187.5, and 250 kg ha^-1^, respectively.

*M_0_*, no straw returning; *M_1_*, straw returning.

^*^ *p* < 0.05 and ^**^p < 0.01 indicate significant effects.

*F*-value, F-statistic in analysis of variance; M, wheat-straw returning treatment; K, K application; M×K, wheat-straw returning treatment and K application interaction.

### 3.5 Straw decay and soil K balance

**Figure 3:**
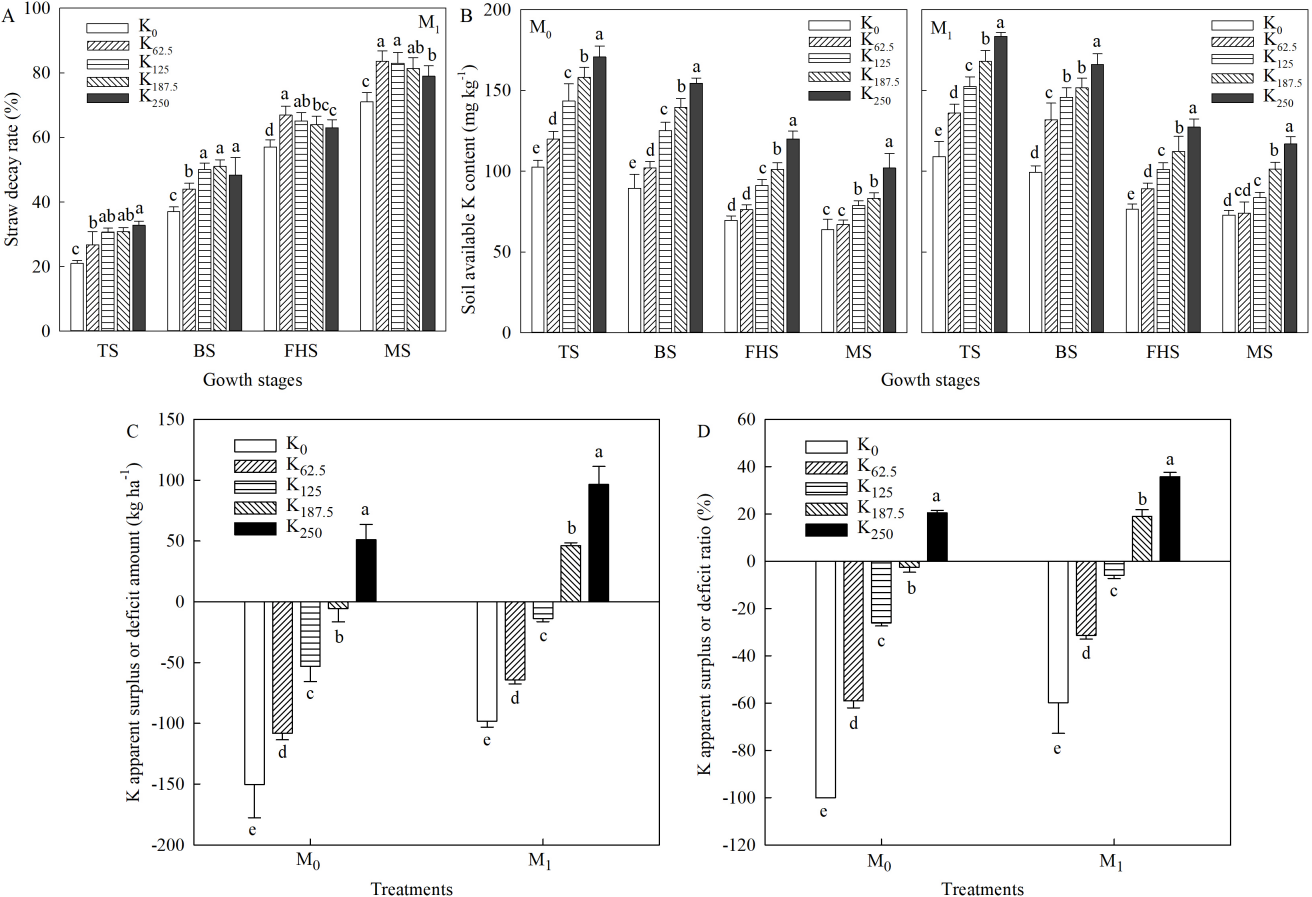
Effects of straw returning and K application rates on straw decay rate **(A)**, soil available K **(B)**, K deficit **(C)**, and surplus ratio **(D)** during the main growth stages of direct-seeded rice. Different lowercase letters under the same straw returning method in the same column indicate significant differences at the 5% level in different K rates. M_0_, no straw returning; M_1_, straw returning; TS, tillering stage; BS, boot stage; FHS, full-heading stage; MS, maturity stage. K_0_, K_62.5_, K_125_, K_187.5_, and K_250_ represent the elemental K fertilizer rates: 0, 62.5, 125, 187.5, and 250 kg ha^-1^, respectively.

K released via straw decomposition is the primary source of soil K replenishment. As shown in **Figure 3A**, the straw decay rate under wheat-straw returning reached 73.88%–83.51% by maturity as rice growth progressed. Significant (*p* < 0.05) differences in decay rate were detected among K treatments across all growth stages. At tillering and jointing, 0–62.5 kg K ha^-1^ could not meet the K demand of straw-decomposing microorganisms, reducing decay rate by 3.98%–11.78% compared with other K treatments. Conversely, the high soil available K content under 250 kg K ha^-1^ (**Figure 3B**) also inhibited straw decay at the full-heading and maturity stages.

At the same K application rate, across all measured growth stages, soil available K content under the M_1_ treatment was 6.00%–29.31% higher than that under the M_0_ treatment (**Figure 3B**), indicating that wheat-straw returning enhances soil available K supply during the middle and late growth stages. Across all straw returning treatments, soil available K content increased progressively with increasing K application rate and peaked at 250 kg K ha^-1^. Within the same straw returning treatment, the available K difference between 125 and 187.5 kg K ha^-1^ narrowed with growth under M_0_ but widened under M_1_, which indirectly verifies the significant buffering effect of wheat-straw returning on soil available K content.

In addition, at the same K application rate, the K apparent surplus/deficit amount and ratio were 39.25–52.13 kg K ha^-1^ and 15.30%–40.18% higher in M_1_ than in M_0_, respectively (**Figures 3C, D**). For the M_0_ treatment, 187.5 kg K ha^-1^ nearly maintained soil K balance, with a small apparent deficit of 5.95 kg ha^-1^ (ratio of − 2.57%). By contrast, 125 kg K ha^-1^ was sufficient for K balance in the M_1_ treatment: although this rate caused an apparent deficit of 13.75 K ha^-1^ (− 5.99%), the gap can be offset by K released from full decomposition of the remaining 20% of wheat-straw to achieve overall soil K equilibrium.

### 3.6 Rice quality

Head rice rate, chalkiness, and taste value are key indicators for evaluating rice quality. Wheat-straw returning, K application, and their interaction exerted significant (*p* < 0.05) or extremely significant (*p* < 0.01) effects on all tested rice quality indexes (**Table 6**). The magnitude of the regulatory effect of the K application rate on each quality index was significantly larger than that of wheat-straw returning. Under the same K application rate, brown rice rate, head rice rate, and taste value in the M_1_ treatment were 0.04%–3.64% higher than those in M_0_, while chalkiness rate and chalkiness degree were 0.05%–2.04% lower. Across all combinations of wheat-straw returning and K application, M_1_K_125_ achieved the highest brown rice rate, head rice rate, and taste value, which were 0.48%–1.15%, 0.54%–3.64%, and 0.29–5.49 higher than those in all other treatments, respectively.

**Table 6:** Effects of straw returning and K application rates on rice quality of direct-seeded rice.

Treatments	Brown rice rate (%)	Head rice rate (%)	Chalkiness rate (%)	Chalkiness degree (%)	Taste value	
					2021	2022
M_0_						
K_0_	74.82 b	60.42 b	18.11 ab	6.60 a	79.67 c	79.02 d
K_62.5_	75.03 ab	61.22 a	17.54 bc	6.11 b	80.33 bc	81.10 c
K_125_	75.35 ab	61.62 a	17.09 c	6.46 a	81.67 b	83.77 b
K_187.5_	75.61 a	61.64 a	15.94 d	4.50 c	83.67 a	84.51 a
K_250_	74.96 b	60.64 b	18.91 a	6.37 ab	82.00 ab	83.21 b
Average	75.15	61.11	17.52	6.01	81.47	82.32
M_1_						
K_0_	76.96 b	61.18 c	17.91 a	5.45 a	80.00 c	80.76 d
K_62.5_	76.94 b	62.11 b	16.56 b	4.70 b	81.33 bc	82.14 c
K_125_	78.09 a	64.82 a	15.30 c	4.42 b	84.00 a	85.90 a
K_187.5_	77.61 ab	64.28 a	15.72 c	4.45 b	83.71 ab	85.26 a
K_250_	77.52 b	63.26 b	17.44 a	5.77 a	82.33 ab	84.17 b
Average	77.42	63.13	16.59	4.94	82.27	83.65
*F*-value						
M	5.94^*^	6.12^*^	5.45^*^	6.07^*^	4.39^*^	5.35^*^
K	6.27^*^	7.98^*^	8.92^**^	8.40^*^	12.53^**^	21.05^**^
M × K	9.01^**^	10.66^**^	15.77^**^	3.96^*^	3.89^*^	4.14^*^

Different lowercase letters under the same straw returning method in the same column indicate significant differences at the 5% level in different K rates. K_0_, K_62.5_, K_125_, K_187.5_, and K_250_ represent the elemental K fertilizer rates: 0, 62.5, 125, 187.5, and 250 kg ha^-1^, respectively.

*M_0_*, no straw returning; *M_1_*, straw returning.

^*^ *p* < 0.05 and ^**^p < 0.01 indicate significant effects.

*F*-value, F-statistic in analysis of variance; M, wheat-straw returning treatment; K, K application; M×K, wheat-straw returning treatment and K application interaction.

### 3.7 Relationships between photosynthetic production and K translocation with yield, rice quality, and KUE

**Figure 4:**
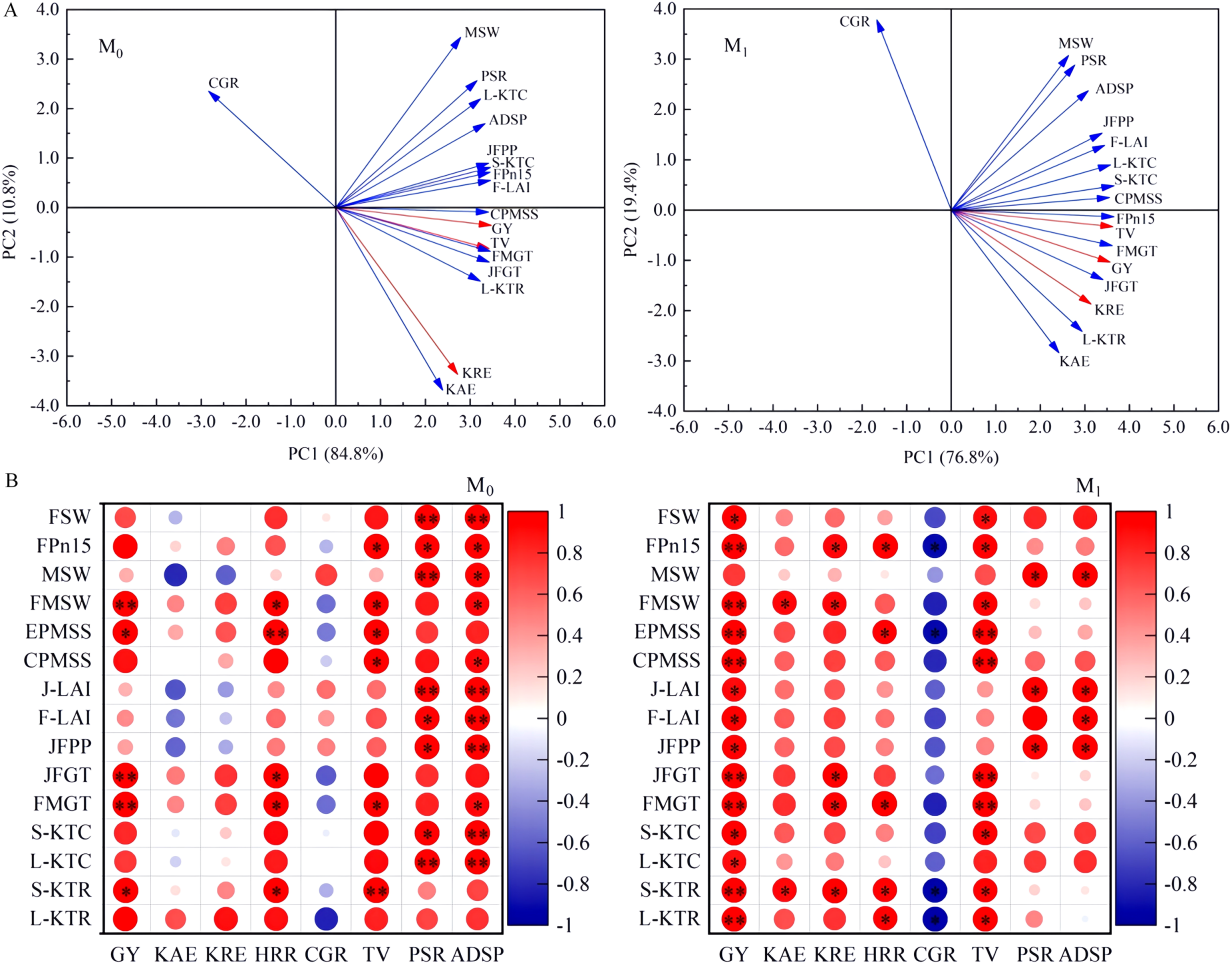
Principal component analysis **(A)** and heat map of person correlation **(B)** in yield, rice quality, and KUE with photosynthetic production and K translocation of direct-seeded rice. M_0_, no straw returning; M_1_, straw returning; GY, grain yield; KAE, K agronomic efficiency; KRE, K physiological efficiency; HRR, head rice rate; CGR, chalkiness rate; TV, taste value; PSR, K apparent surplus/deficit ratio; ADSP, K apparent surplus/deficit amount; FSW, stem-sheath dry matter weight at the full heading; MSW, stem-sheath dry matter weight at the maturity; FMSW, dry matter weight from full heading to maturity; EPMSS, stem-sheath matter translocation rate from full heading to maturity; CPMSS, stem-sheath matter translocation contribution rate from the full heading to maturity stage. J-LAI, LAI at the jointing stage; F-LAI, LAI at the full-heading stage; FPn15, net photosynthetic rate of flag leaves at 15 days after the full heading; JFPP, photosynthetic potential from jointing to full heading; JFGT, population growth rate from jointing to full heading; FMGT, population growth rate from full heading to maturity; S-KTC, K translocation amount in stem sheaths; L-KTC, K translocation amount in leaves; S-KTR, K translocation contribution rate in stem sheaths; L-KTR, K translocation contribution rate in leaves. ^*^p < 0.05 and ^**^p < 0.01 indicate significant effects.

Principal component analysis (**Figure 4A**) showed that, for the M_0_ and M_1_ treatments, the first two principal components cumulatively explained 95.6% and 96.2% of the total variation, respectively, in grain yield, KRE, chalkiness rate, head rice rate, taste value, and apparent K surplus/deficit ratio across different K application rates. Across all K application rates, compared with the M_0_ treatment, the yield formation, rice quality, and KUE of the M_1_ treatment clearly illustrated the coordinated improvement of high-quality, high-yield, and high-KUE performance.

Correlation analysis (**Figure 4B**) showed that under all K application rates in the M_0_ treatment, grain yield (GY), taste value (TV), and head rice rate (HRR) were significantly positively correlated (*r* = 0.54^*^–0.90^**^) with four traits measured from FMSW, EPMSS, FMGT, and stem-sheath K translocation contribution rate (S-KTR). FMSW, EPMSS, FMGT, and S-KTR showed no significant correlation with KAE, KRE, and chalkiness rate (CGR). In addition, FSW, MSW, net photosynthetic rate of flag leaves at 15 days after full heading (FPn15), J-LAI, F-LAI, stem-sheath K translocation amount (S-KTC), and leaf K translocation amount (L-KTC) were all significantly positively correlated (*r* = 0.56^*^–0.92^**^) with apparent K surplus/deficit ratio (PSR) and apparent K surplus/deficit amount (ADSP). These results indicated that soil K surplus or deficit directly affected these physiological traits under the M_0_ treatment and subsequently influenced the aboveground growth of rice.

A further correlation analysis (**Figure 4B**) showed that across all K application rates in the M_1_ treatment, GY, TV, and HRR were significantly positively correlated (*r* = 0.49^*^–0.93^**^) with FPn15, EPMSS, FMGT, S-KTR, and leaf K translocation contribution rate (L-KTR). Moreover, higher values of FPn15, FMSW, FMGT, and S-KTR benefited improvements in KAE and KRE. In addition, FPn15, EPMSS, S-KTR, and L-KTR were all significantly negatively correlated with CGR (*r* = − 0.46^*^ to − 0.57^*^). Moreover, this effect was not affected by soil K deficiency, and S-KTR could serve as a key indicator for high-yield, quality improvement, and efficiency enhancement of direct-seeded rice. In addition, a comprehensive evaluation was conducted based on indicator weights and membership function values (**Table 7**). The results showed that the M_1_K_125_ treatment obtained the highest overall evaluation score.

**Table 7:** Comprehensive evaluation and ranking of principal components of straw returning and K application rates.

Treatments	Integrated index value		Subordinate function value		Comprehensive score	Rank
	CI1	CI2	μ(*X*_1_)	μ(*X*_2_)	*D*-value	
M_0_K_0_	− 1.65	− 0.14	0.00	0.41	0.06	10
M_0_K_62.5_	− 0.79	− 0.53	0.29	0.30	0.29	8
M_0_K_125_	− 0.07	− 0.24	0.53	0.38	0.50	6
M_0_K_187.5_	0.75	0.20	0.80	0.51	0.76	3
M_0_K_250_	0.25	1.49	0.63	0.89	0.67	5
M_1_K_0_	− 1.17	0.12	0.16	0.49	0.21	9
M_1_K_62.5_	− 0.17	− 0.70	0.49	0.24	0.46	7
M_1_K_125_	1.36	− 1.54	1.00	0.00	0.85	1
M_1_K_187.5_	0.94	− 0.13	0.86	0.41	0.80	2
M_1_K_250_	0.50	1.87	0.71	1.00	0.75	4
Weight			0.85	0.15		

K_0_, K_62.5_, K_125_, K_187.5_, and K_250_ represent the elemental K fertilizer rates: 0, 62.5, 125, 187.5, and 250 kg ha^-1^, respectively.

*M_0_*, no straw returning; *M_1_*, straw returning.

## 4 Discussion

### 4.1 Effect of wheat-straw returning and K management on yield formation and economic benefit in direct-seeded rice

Rice yield formation is closely associated with population photosynthetic characteristics and the capacity for dry matter accumulation and translocation (Bassuony, 2019; Iqbal et al., 2019; Sun et al., 2024). Grain yield is determined by three core yield components: effective panicle number, filled spikelets, and 1,000-grain weight (Tian et al., 2022). However, current studies have reached inconsistent conclusions regarding the effects of straw returning on yield components of direct-seeded rice (Li et al., 2021). Previous studies (Ma et al., 2023; Guo et al., 2023a; Huo et al., 2024) have demonstrated that straw returning promotes nutrient absorption in the roots of direct-seeded rice, effectively increases total spikelets and filled grains, and ultimately improves yield stability. Consistent with these findings, our study shows that under the same K fertilization treatment, wheat straw returning significantly increases total spikelet number and further boosts grain yield while maintaining high filled grains and 1,000-grain weight (**Table 2**). On the other hand, Ran et al. (2023) proposed that straw returning optimizes grain filling by increasing LAI across key growth stages of direct-seeded rice, which in turn increases 1,000-grain weight and promotes yield. However, our results do not support this mechanism. Relative to M_0_, M_1_ significantly increased grain yield but did not produce a significant increase in jointing-stage LAI (**Table 3**), nor did it have a significant effect on 1,000-grain weight (**Table 2**). Our results confirm that increased total spikelet number under wheat-straw returning is the key driver of yield improvement in direct-seeded hybrid *indica* rice.

K fertilizer can increase the seed-setting rate and 1,000-grain weight to achieve high yields. However, there are also disputes regarding the influence of increasing K fertilizer application on the yield components of direct-seeded rice at present (Kumar et al., 2015; Ullah et al., 2019; Zhang et al., 2021a). Kumar et al. (2015) reported that K fertilizer primarily increased the number of effective panicles but had no significant effect on filled spikelets or 1,000-grain weight. Conversely, Ullah et al. (2019) observed increases in total spikelets and filled grains upon K fertilization, with occasional inhibition of filled spikelet and panicle formation. In contrast, our results reveal a significant synergistic effect between wheat-straw returning and K fertilization. The yield gain achieved under the M_1_K_125_ treatment is primarily attributed to optimized source–sink relationships. Specifically, this treatment improved *P*_n_ after full heading (**Figure 1**) and enhanced overall canopy photosynthetic capacity: it increased population photosynthetic potential as well as population growth rate in both the jointing to full-heading stage and the full heading to maturity stage (**Table 3**). It also significantly improved the export and contribution of stem-sheath stored matter (**Table 4**). Collectively, these changes facilitate more efficient translocation of assimilates to developing panicles, which in turn drives improvements in yield components and ultimately increases final grain yield. These results further indicate that integrating straw returning with K fertilization offers a greater yield advantage than K fertilization alone.

Notably, the M_1_K_125_ treatment recorded a significant 6.44%–9.01% (575.4–802.1 kg ha^-1^) yield increase compared with the optimal K treatment without wheat-straw returning (M_0_K_187.5_, 187.5 kg K ha^-1^). Based on the rice market price of 2.60 yuan kg^-1^, this yield increase translates to an additional output value of 1,496.6–2,085.5 yuan ha^-1^. Meanwhile, this treatment reduced K input from 187.5 to 125 kg K ha^-1^, saving 62.5 kg K ha^-1^. Calculated at a market price of 3,200 yuan per tonne for KCl (49.8% elemental K content), this K reduction corresponds to a fertilizer cost saving of 401.2 yuan ha^-1^. Combining the additional output revenue and cost savings, the total net income gain reaches 1,897.6–2,487.1 yuan ha^-1^. This clearly demonstrates that wheat straw returning reduces the optimal K application rate required to achieve maximum grain yield in direct-seeded rice.

### 4.2 Effect of wheat-straw returning and K management on KUE and rice quality in direct-seeded rice

Rational utilization of K derived from wheat straw returning cannot only improve KUE of rice but is also closely associated with improved rice quality (Jin et al., 2020; Huo et al., 2024). Results from this experiment showed that, compared with treatments without wheat-straw returning, KAE and KRE were significantly improved in wheat-straw returning treatments (**Table 5**). This confirms that K released from wheat straw can be effectively absorbed by rice plants. When combined with supplementary K fertilizer application, this practice can effectively reduce the required K application rate while further improving KAE and KRE. This further demonstrates that integrating wheat-straw returning with K fertilization can reduce K fertilizer inputs and achieve high-efficiency and cost-effective rice production. In addition, previous studies (Du et al., 2021) have reported that K accumulation in rice is mainly associated with the translocation of photosynthetic assimilates, and there is a positive correlation between K uptake and grain yield. Conversely, our results showed that grain yield increased first and then decreased with increasing K accumulation in rice plants (**Figure 2**, **Table 2**). This divergence from previous findings is likely because excessively high K application rates reduce rice plants’ demand for K released from straw, leading to luxury K uptake. While luxury uptake increases total K accumulation in plants, it is not conducive to the translocation of K to grains (Ye et al., 2020; Ran et al., 2023). Consistent with previous findings (Xue et al., 2015), K primarily accumulates in the stem sheath of rice plants and is transported from the stem sheaths to panicles after heading. However, our study found that during the grain-filling stage, the K translocation contribution rate of leaves in direct-seeded rice was much higher than that of the stem sheaths. Moreover, under wheat-straw returning, the K translocation contribution rate of leaves increased significantly with increasing K application rate (**Table 5**). This indicates that K from all plant organs can be effectively transported to the panicle, which promotes postheading dry matter accumulation in panicles and thus increases grain yield. When K fertilizer input is excessive, however, both the K translocation contribution rate and translocation amount in leaves and stem sheaths decrease. Therefore, K fertilizer application for direct-seeded rice under wheat-straw returning must be controlled within a reasonable range (Ye et al., 2020).

Regarding KUE, previous studies have reported that KAE increases with K application rate up to a threshold, then declines (Xue et al., 2015; Zhang et al., 2021a). Consistent with these findings, our results showed that in both wheat-straw returning and no straw returning treatments, KAE and KRE of direct-seeded rice followed the same trend of an initial increase followed by a decline as K input increased, and both parameters were significantly higher under wheat-straw returning than no straw returning (**Table 5**). This can be attributed to the stronger promoting effect of the straw-K fertilizer interaction on KUE than that of single K fertilization (Ullah et al., 2019; Zhang et al., 2021b). Regarding the effect of K fertilization on rice quality, existing studies have confirmed that K application significantly affects milling quality (head rice rate) and appearance quality (chalkiness rate, chalkiness degree), and moderate K addition improves taste value (Zhang et al., 2021a; Chen et al., 2023). In the present study, under wheat-straw returning combined with K fertilization, brown rice rate, head rice rate, and taste value all increased initially, then declined with increasing K input, while chalkiness showed the opposite trend. These results confirm that appropriate K application under wheat-straw returning can synergistically improve KUE and rice quality. However, excessive K application promotes protein synthesis in grains and reduces taste value (Chen et al., 2021; Guo et al., 2023b). Compared with previous reports that N fertilizer exerts greater effects on chalkiness in the super rice variety Fyou 498 under direct-seeded conditions (Chen et al., 2023; Sun et al., 2024), our results further reveal that combined K fertilization has a more prominent effect on improving rice taste value. This indicates that additional K input outperforms N input in improving the grain quality in direct-seeded rice, and the optimal treatment in this experiment was wheat-straw returning combined with 125 kg K ha^-1^ (**Table 7**).

### 4.3 Effect of wheat-straw returning and K management on soil K balance

Crop straw stores 75%–80% of total absorbed K in crop production (Das et al., 2021; Lodi et al., 2022). Straw returning can effectively offset crop K demand, improve soil K supply capacity, and maintain soil K balance (Singh et al., 2018; Zhang et al., 2021b). Consistent with this function, our results showed that after decomposing incorporated wheat-straw (**Figure 3A**), straw returning significantly increased soil available K content (**Figure 3B**) and reduced its fluctuation across the entire growth period of direct-seeded rice compared with no straw returning. An earlier study (Ocio et al., 1991) reported that, although straw returning increases soil available K content, soil K still remains in deficit after rice harvest, despite a significant reduction in K loss. Our results differ from these previous findings: the apparent soil K deficit was significantly lower under wheat-straw returning than under no straw returning. However, apparent soil K balance shifted to surplus when 250 kg K ha^-1^ was applied without straw returning or when 187.5 kg K ha^-1^ was applied with wheat-straw returning (**Figure 3C**). This can be attributed to excessive K input exceeding the K demand of direct-seeded rice, leading to K supply in excess of crop requirement (Ye et al., 2020). Wheat-straw returning combined with 187.5 kg K ha^-1^ facilitated the transition of soil K from deficit to surplus and maintained soil K balance. Notably, although 125 kg K ha^-1^ under wheat-straw returning resulted in an apparent K deficit of 13.75 kg K ha^-1^, approximately 20% of wheat-straw remained undecayed by rice harvest in this experiment. Full decomposition of the remaining straw would completely maintain K balance in rice-wheat rotation systems.

Screening indicators that directly assess KUE, rice quality, and yield are critical for the coordinated improvement of crop yield and quality (Peng et al., 2002). Previous studies have shown that across different K application rates, dry matter accumulation, maturity-stage K accumulation, and K recovery efficiency are closely associated with high yield, high quality, and high KUE in rice (Chen et al., 2024). Additional work has also confirmed that under straw returning, rice LAI and soil available K content are closely correlated with rice yield and KUE (Ullah et al., 2019; Luo et al., 2024). Our principal component and correlation analyses (**Figure 4**) further revealed that under wheat-straw returning, multiple traits promote the simultaneous improvement of yield, quality, and KUE in direct-seeded rice: photosynthetic potential from jointing to full heading, flag leaf net photosynthetic rate at 15 days after full heading, and a set of translocation traits from full heading to maturity, including stem-sheath dry matter contribution rate, stem-sheath K translocation amount, and stem-sheath/leaf K translocation contribution rate. In particular, stem-sheath K translocation contribution rate from full heading to maturity showed a strong significant correlation (*r* = 0.50^*^–0.96^**^) and was identified as the core influencing factor. Therefore, this indicator can also serve as a unified diagnostic indicator for the coordinated improvement of yield, rice quality, and KUE in direct-seeded rice.

## 5 Conclusion

Wheat-straw returning and K fertilizer management exhibited a significant interaction for grain yield, grain quality, and KUE in mechanically direct-seeded rice. The combined application of 5,050–5,440 kg ha^-1^ wheat-straw returning with 125 kg K ha^-1^ significantly improved the stem-sheath K translocation contribution rate from full heading to maturity, thereby increasing grain yield and KUE, improving grain quality, and maintaining soil K balance. This optimized strategy has been further tested across different agroecological regions and has been shown to significantly increase farmers’ income and boost their production enthusiasm.

## Data Availability

The raw data supporting the conclusions of this article will be made available by the authors, without undue reservation.
