# Supplementary material for: Enhancing the yield, quality, and potassium use efficiency of direct-seeded hybrid indica rice through wheat-straw returning combined with potassium fertilizer application
Source: Front Plant Sci. 2026 May 13;17:1839324. doi: 10.3389/fpls.2026.1839324 (PMC13212227; doi:10.3389/fpls.2026.1839324)
Supplement: Supplementary file 1 [file Table1.docx]

***Supplementary Material***

**Supplementary TABLE 1** Analysis of variance for grain yield, KUE, and taste value of rice between years and straw returning and K fertilizer application (*F* values) (2021–2022)

| Analysis of variance | Grain yield | Total spikelets | KAE | KPE | Taste value |
| --- | --- | --- | --- | --- | --- |
| Year (Y) | 1.84^ns^ | 2.53^ns^ | 2.31^ns^ | 1.31^ns^ | 0.98^ns^ |
| Y × Straw treatment (M) | 0.38^ns^ | 1.21^ns^ | 0.15^ns^ | 0.09^ns^ | 0.72^ns^ |
| Y × K treatment (K) | 0.25^ns^ | 1.42^ns^ | 0.41^ns^ | 0.33^ns^ | 0.18^ns^ |
| Y × M × K | 0.67^ns^ | 0.75^ns^ | 0.52^ns^ | 0.48^ns^ | 0.84^ns^ |

KAE: K agronomic efficiency; KPE: K physiological efficiency. ns denote non-significance (*P* > 0.05).
